# Xiangshao Granule Exerts Antidepressive Effects in a Depression Mouse Model by Ameliorating Deficits in Hippocampal BDNF and TrkB

**DOI:** 10.1155/2013/309262

**Published:** 2013-12-03

**Authors:** Yi Chen, Jie Liu, Xiaoting Wu, Edouard Collins Nice

**Affiliations:** ^1^Department of Gastrointestinal Surgery, State Key Laboratory of Biotherapy, West China Hospital, Sichuan University, Chengdu 610041, China; ^2^Monash University, Department of Biochemistry and Molecular Biology, Clayton, Victoria 3800, Australia; ^3^Visiting Professor, West China Hospital, Sichuan University, Chengdu 610041, China

## Abstract

This study explores the therapeutic effects of Xiangshao granules in a mouse depression model and examines the potential molecular mechanisms involved. After 21 consecutive days of chronic stress challenge, all mice were divided into three groups: control group, depression group, and Xiangshao granule treatment group. On the 22nd day, rats in the Xiangshao granule treatment group received Xiangshao granules via gastrogavage for 3 consecutive weeks. Depression group mice showed a significant reduction of crossings (*P* < 0.01) but not rearings (*P* < 0.05). Serum CRH, CORT, and ACTH levels were significantly increased in depression mice compared with control (*P* < 0.05) and the expression levels of hippocampal BDNF and TrkB were reduced in the model group (*P* < 0.05). However, Xiangshao granule treatment remarkably rescued the decrease in the body weight (*P* < 0.05), increased crossings in the open field test (*P* < 0.05), upregulated the expression of hippocampal BDNF and TrkB expression, and reduced the serum CRH, CORT, and ACTH concentrations compared with the depression group (*P* < 0.05). Collectively, these results demonstrated that Xiangshao granule could effectively induce antidepressive effects in the depression mouse model by ameliorating the expression of hippocampal BDNF and TrkB.

## 1. Introduction

Depression constitutes one of the major causes of disability and is the 4th leading cause of the global burden of disease, with a lifetime prevalence of up to 17% [1]. Although antidepressant medications represent the mainstay therapy for depression, almost one-quarter of the patients fail to respond to these therapeutics [2]. Attempts are therefore being made to seek alternative medication.

Chinese traditional medicine treatment for patients suffering depression has more than a thousand years of history. It has proved efficacious and thus is attracting increasing attention from scientists working in this field.

Xiangshao granule, which is made from the root of the herbaceous peony, *Cyperus*, and ligustrazine, and other natural products [3], has been demonstrated to exert therapeutic effects for premenstrual tension syndrome [4, 5]. Expression of *μ*-opioid receptor in brains of premenstrual tension syndrome Gan-qi invasion rats was regionally specific, and administration of Xiangshao granule showed corresponding regulatory effects [4]. It has been reported that the binding potential of the *μ*-opioid receptor is significantly lower in patients with major depressive disorders relative to nondepressed controls [5]. However, whether Xiangshao granule could also improve the outcome for depression patients and, if so, the underlying mechanisms involved remains unclear.

Previous research has demonstrated that neuronal dysfunction and apoptosis are closely related to development of depression [6]. Brain-derived neurotrophic factor (BDNF), which is a member of the neurotrophic factor family [7], can promote neuronal formation and regulate synaptic plasticity by activation of its cognate high affinity receptor tyrosine kinase B (TrkB) to function as an antidepressant [8]. In this study, we have evaluated the antidepressant effects of Xiangshao granule in a chronic stress challenged mouse model and have explored its potential impact on hippocampal BDNF and TrkB expression.

## 2. Materials and Methods

### 2.1. Animal Models

**Figure 1:**
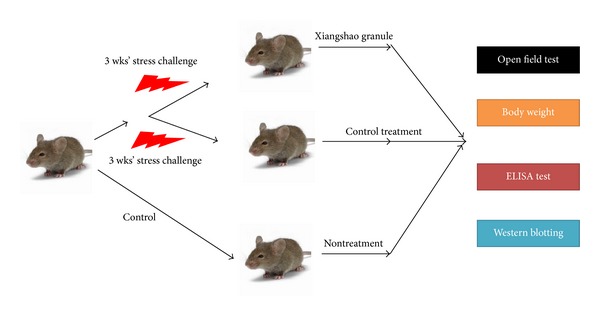
A schematic of the animal studies displaying the three animal groups and their program and regimen in terms of stress periods and treatments.

The schematic of animal studies is shown in Figure 1. A total of 21 pathogen-free, Balb/c mice were kept in an air-conditioned room with a 12 h light/dark cycle with free access to food and water except when animals were subjected to deprivation stressors. The animals were allowed to acclimatize to the environment for 2 weeks (control phase). The chronic mild stress procedure used was as described previously [9]. Briefly, the stress regime consisted of the following elements: forced bath, water and/or food deprivation, pairing with another stressed animal in wet sawdust, housing in wet sawdust, reversal of the light/dark cycle, and housing in constant illumination or darkness each for a period ranging from 10 min to 24 h, in a schedule that lasts for 3 weeks, and was repeated thereafter weekly. Stressors were administered throughout the experiment, could occur at any time of day (or night), and were applied each for a period of between 8 and 24 h. Their sequence was random, in order to be completely unpredictable to the animal. This study was approved by the Institutional Ethics Committee of Sichuan University.

### 2.2. Open Field Test

An open field test was conducted as previously described [10]. Briefly, this test was conducted in a square white Plexiglas open field (each side 1.22 m, height 45 cm). The apparatus floor was divided into 16 equal squares for assessing locomotion. The field was cleaned thoroughly between tests. Each mouse was tested for 6 minutes.

### 2.3. Xiangshao Granule Treatment

Xiangshao granule treatment was as described previously in a premenstrual syndrome study [4]. After 3 weeks' chronic mild stress challenge, mice in the treatment group received treatment at a dosage of 10 mg/kg body weight per day through gastrogavage administration.

### 2.4. Measurement of Serum CRH, CORT, and ACTH Levels

Serum levels of CRH, CORT, and ACTH in each experimental group were measured by using the CRH, CORT, and ACTH enzyme-linked immunosorbent assay (ELISA) kit. Detailed ELISA procedures were described elsewhere [11].

### 2.5. Western Blotting

After the behavioral tests were completed, seven rats in each group were sacrificed by decapitation to obtain samples for Western blot analysis. The hippocampus was dissected and put into chilled tubes treated with enzyme inhibitors. This tissue was homogenized to a fine powder using a Waring blender and Western blot analysis performed as previously reported [12], using primary antibodies for rabbit BDNF, TrkB, and *β*-actin at 1 : 1000 dilution (Santa Cruz Biotech Inc., CA, USA). Subsequently, membranes were washed and treated with appropriate secondary antibodies conjugated to horseradish peroxidase for 2 h. Immunoreactivity was visualized by enhanced chemiluminescence reagents (Millipore, WBKLS0500). *β*-Actin was used as an internal control.

### 2.6. Statistical Analysis

Values are presented as mean ± SD. Unpaired *t*-test or Pearson correlation test was used to compare quantitative variables. Pearson *χ* ^2^ test or Fisher's exact test was applied to compare qualitative variables. Analysis was performed with SPSS 13.0 for Windows (SPSS Inc., Chicago, IL, USA). *P* < 0.05 was considered statistically significant.

## 3. Results

### 3.1. Effects of Xiangshao Granule on Body Weight

**Figure 2:**
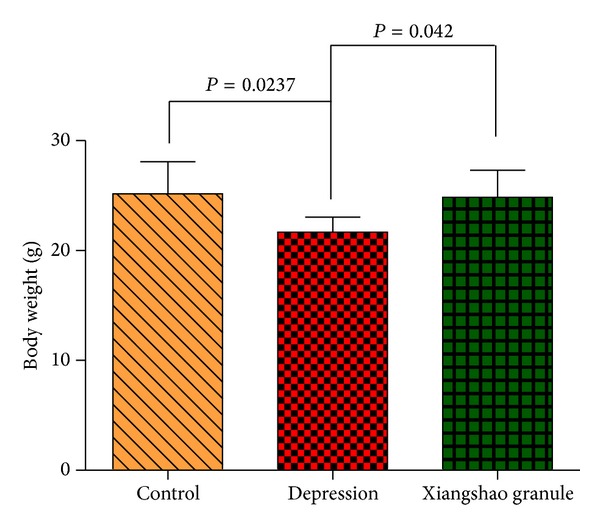
The effect of Xiangshao granule on mice body weight for control mice, mice following chronic stress challenge (depression model), and depressed mice treated with Xiangshao granules.

At the beginning of the experiment, no significant difference in body weight was observed among groups (*P* > 0.05, data not shown). After stress challenge, a significant difference was observed between groups, with the stress (depressed) group showing a significant decrease in body weight compared to the normal mouse (*P* = 0.0237) (Figure 2). However, Xiangshao treatment effectively rescued the decrease in body weight in the depressed mouse (*P* = 0.042) (Figure 2).

### 3.2. Effects of Acupuncture Treatment on Open Field Test

**Figure 3:**
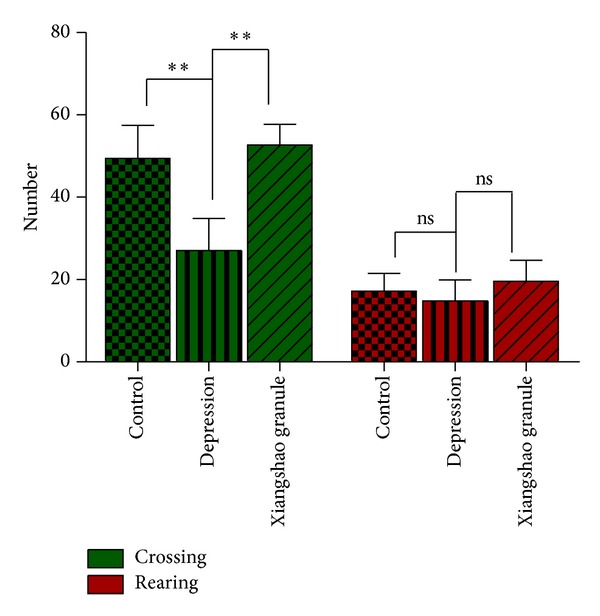
The effect of Xiangshao granules on locomotor activity of mice in the open field test. Groups analysed were as in Figure 1. as not significant, *P < 0.05, **P < 0.01.

The open field test was used to study exploratory and locomotor activity [13, 14]. As seen in Figure 3, significant differences between groups were observed using this test in the number of crossings after stress challenge (*P* < 0.01). Compared with control, depression mice showed a significant reduction of crossings (*P* < 0.01) but not rearings (*P* > 0.05). Xiangshao granule treatment significantly improved the locomotor activity, which was decreased by chronic stress challenge (*P* < 0.01). These results suggested that Xiangshao granule may exert antidepression activities in the depression mouse model.

### 3.3. Effects of Xiangshao Granule on the Serum Levels of CRH, CORT, and ACTH in Mice

**Figure 4:**
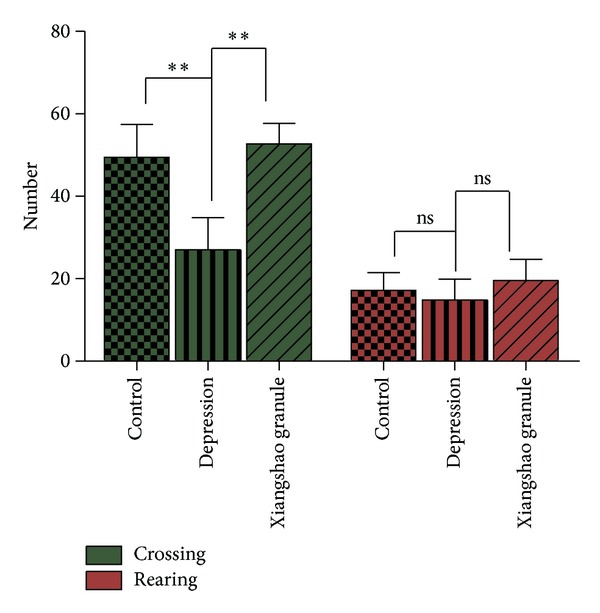
The serum levels of CRH, ACTH, and CORT for mice in the groups shown in Figure 1. *P < 0.05, **P < 0.01.

Previous studies have shown that increased serum levels of CRH, CORT, and ACTH are associated with an increased risk of depression, suicide, and certain types of aggression [11]. We therefore examined the serum levels of CRH, CORT, and ACTH in each test cohort using ELISA. The serum levels of CRH, CORT, and ACTH were significantly increased in the depressed mouse model compared with the control group (*P* < 0.05) (Figure 4). Interestingly, Xiangshao granule treatment significantly decreased the serum levels of CRH, CORT and ACTH in the depression mouse model (*P* < 0.05). These results further suggested that Xiangshao granule could possess antidepression activity by reducing serum CRH, CORT, and ACTH levels.

### 3.4. Downregulation of Hippocampal BDNF and TrkB Expression after Chronic Mild Stress Challenge

**Figure 5:**
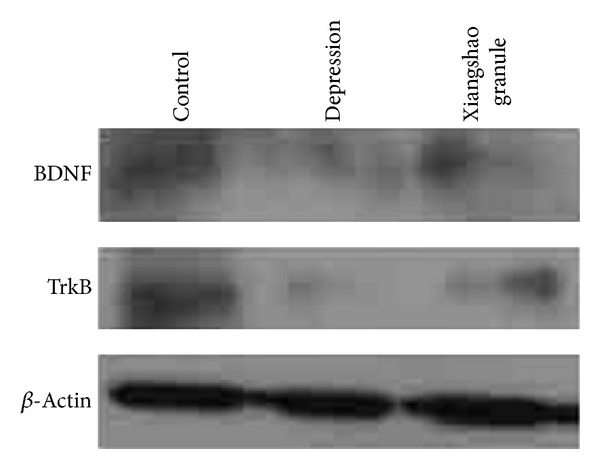
Western blot analysis showing the effects of Xiangshao granule treatment on hippocampal BDNF and TrkB protein expression in mice from the groups indicated. *β*-Actin serves as internal control.

Previous studies have demonstrated that many different types of chronic stress regimes decrease the expression of BDNF, the most abundant brain neurotrophin, in the hippocampus [7]. In addition, researchers have found that other types of stress, including chronic unpredictable stress, swim stress, footshock, and maternal deprivation, could also decrease the expression of hippocampal BDNF [15]. In this study, we first examined whether chronic mild stress decreased the BDNF expression in the mouse hippocampus. As shown in Figure 5, BDNF expression was significantly decreased in the hippocampus of mice that received a 21-day regime of chronic mild stress. TrkB is a high affinity catalytic receptor for several “neurotrophins,” which are small protein growth factors that induce survival and differentiation of distinct cell populations. TrkB is considered as the preferred receptor of BDNF [15]. Hence, we also examined the expression levels of TrkB in the hippocampus of chronic mild stress-challenged mice. Data showed that chronic mild stress also decreased the TrkB expression levels in the hippocampus of mice as predicted (Figure 5). These results demonstrated that chronic mild stress significantly downregulated BDNF and TrkB expression in the mouse hippocampus and thereby led to suppression of the BDNF-TrkB signaling pathway in the hippocampus of chronic mild stress-challenged mice.

### 3.5. Regulation of BDNF and TrkB Expression by Xiangshao Granule Treatment

As described previously, Xiangshao granule treatment partly restored locomotor activity in the depressed mouse model, suggesting that Xiangshao granule may have antidepression effects [4]. Since the BDNF-TrkB signaling pathway plays an essential role in antidepression treatment, and chronic mild stress significantly decreased both BDNF and TrkB expression [7], we investigated whether Xiangshao granule treatment could restore BDNF and TrkB expression levels in the chronic mild stress mouse model. Western blot analysis of hippocampal samples showed that Xiangshao granule treatment induced significant upregulation of total BDNF protein levels (Figure 5). In addition, western blot analysis also showed that the TrkB level was much higher than that of the untreated depressed group (model group) (Figure 5). These results indicated that Xiangshao granule treatment increased expression of BDNF and its receptor TrkB and thus activated the BDNF-TrkB signaling pathway in the hippocampus of depressed mice.

## 4. Discussion

The standard pharmacological treatment for depression is antidepressants, which can reduce symptom severity and prevent relapses. Unfortunately, however, antidepressants also have several drawbacks including limited efficacy and undesirable side effects including dizziness, sleepiness, and constipation, with not all patients responding to currently available pharmacological treatments. This strongly impairs both therapeutic use and efficacy for many depression patients, and novel agents with fewer side effects are therefore urgently needed. Xiangshao granule is a well-documented regimen of Chinese traditional medicine that consists of the root of the herbaceous peony, *Cyperus*, ligustrazine, and other natural products and which has been shown to alleviate premenstrual syndrome [4]. In this study, we report that Xiangshao granule also exerts antidepression activity by increasing the expression of hippocampal BDNF and TrkB expression in a depression mouse model. This antidepressive effect of Xiangshao granule appeared to be related to decreased serum levels of CRH, CORT, and ACTH in the mouse depression model used.

The pathogenesis of depression is associated with a variety of factors including both psychological and neuroendocrine components [16]. Although the Monoamine Hypothesis is the generally accepted pathogenic mechanism [16], it still does not fully explain the pathophysiology of depression. In recent years, nerve nutrition theory has become the focus of interest in the study of depression pathogenesis [16]. This theory proposes that mood disorders are primarily caused by abnormal neuronetwork adaptability to disturbances in the external environment. However, antidepressant drugs may enhance neuroplasticity and modify neural networks based on outside stimuli.

Accumulating evidence suggests that the cause of depression is related to neural atrophy and necrosis of the hippocampus [17]. The loss of BDNF and TrkB could probably induce hippocampal neuronal atrophy and apoptosis, promoting depression, and may thus be a major pathogenesis of this disease. BDNF, which is synthesized in the neurons and transported to the axonal peripheral, is one of the most important neurotrophic factors in the central nervous system [17] and can stimulate and maintain the growth of new neurons via activation of specific receptors (primarily TrkB). In the central nervous system, BDNF and its receptor TrkB-positive cells are widely distributed in the regions of hippocampus, cortex substantia nigra, and stratum with the hippocampus CA3 region containing the highest content. Stress can significantly decrease protein expression levels of BDNF and its receptor TrkB [17] and leads to atrophy of the brain limbic system structure (such as hippocampus and prefrontal cortex) [17], finally inducing depression. BDNF plays an important regulatory role in the plasticity of hippocampal neuronal structure and function as well as suppression of apoptosis and stabilization of cell proteins [17]. On the other hand, BDNF can promote glutamate (Glu) release through presynaptic receptor signal transduction pathways and participate in the long-term potentiation (LTP) process [18]. When BDNF binds to its high affinity receptor TrkB, it is retrogradely transported to the cell body where it promotes biological effects to enhance synaptic contacts as well as neuronal plasticity, neurotransmission, and neurotrophic factor synthesis leading to control of depression. Other studies have shown that BDNF can also promote the binding of 5-hydroxy tryptamine (5-HT) and 5-HT1A receptors (5-HT1AR) to increase cellular membrane K+ flowing inward, inhibit Glu receptor opening, and weaken Glu induction of Ca^2^+ flowing inward to effectively reduce impairment caused by stress towards hippocampal neurons and protect hippocampal cells [18].

In parallel with BDNF, elevated expression of hippocampal TrkB receptor has been proved to be involved in the therapeutic action of antidepressant treatment in rats and chronic antidepressant treatment has been reported to enhance hippocampal neurogenesis as well as increasing the expression of BDNF and TrkB receptor in animal models [19]. BDNF triggers TrkB receptor-dependent different intracellular signaling pathways and exhibits beneficial effect for the treatment of depression in experimental studies [20]. However, one previous study corroborated that the expression of TrkB could be downregulated following BDNF treatment and the mechanisms underlying involved ubiquitin-proteasome pathway. This explains the phenomenon that although BDNF-TrkB signaling is downregulated in hippocampus according to animal models of stress and/or depressive-like behavior, some studies failed to detect evidence for depression with reduced BDNF levels in meta-analyses [21] or heterozygous mutant *bdnf* +/− mice [22]. However, in contrast to the unclear association with the pathophysiology of depression, the neurotrophin signaling appears to be required for antidepressant activity. In a previous study, drugs that block TrkB signaling pathway can break the antidepressant-like effect of BDNF in rats [23]. Our study found that TrkB and BDNF protein expression increased following Xiangshao granule treatment.

Studies have suggested that increased serum levels of CRH, CORT, and ACTH are associated with an increased risk of depression [18]. Our ELISA results showed increased serum levels of CRH, CORT, and ACTH in the depressed mouse model compared with the control group. However, Xiangshao granule treatment significantly reduced the serum levels of CRH, CORT and ACTH in the depressed mouse model. These results suggest that Xiangshao granule may have antidepression activity by decreasing the serums level of CRH, CORT, and ACTH in depression mouse model.

Collectively, our study demonstrates that Xiangshao granule has a significant antidepressant effect in the mouse depression model with its desired therapeutic effect possibly achieved through increasing hippocampal BDNF and TrkB expression and downregulating serum CRH, CORT, and ACTH levels. Therefore, Xiangshao granule might serve as a novel antidepressant in future clinical practice.

## Abbreviations

ACTH: Adrenocorticotropic hormone
BDNF: Brain-derived neurotrophic factor
CORT: Corticosterone
CRH: Corticotropin-releasing hormone
TrkB: Tropomyosin receptor kinase B.
